# Cardiovascular effects and tolerance of adenosine versus regadenoson during cardiac MRI perfusion testing

**DOI:** 10.1186/1532-429X-17-S1-P86

**Published:** 2015-02-03

**Authors:** Matthew Minor, Joshua Lee, Kevin Steel

**Affiliations:** 1 Cardiology, San Antonio Military Medical Center, Fort Sam Houston, TX, USA; 2 Radiology, San Antonio Military Medical Center, San Antonio, TX, USA

## Background

Patients referred to cardiac MRI (CMR) for suspicion of obstructive coronary artery disease typically undergo vasodilator stress testing using either adenosine or regadenoson. Both of these agents are effective and have been evaluated for stress testing in both the CMR and nuclear medicine setting but the cardiovascular physiologic effects of each of these agents have not been well studied. Despite different pharmacokinetics, stress CMR protocols do not differ between agents, and it is truly unknown if the agents alter ventricular volumes, function and flow differently. In this study, we evaluated the physiologic cardiovascular effects of each of these agents over time in healthy volunteers.

## Methods

We performed a prospective non-randomized controlled trial of 25 healthy volunteers undergoing stress CMR. Vital signs, ventricular volumes, pulmonic and aortic flow were measured at baseline and immediately following infusion of adenosine (140mcg/kg/min IV for 6 minutes) and at 5, 10, and 15 minutes after infusion utilizing a 1.5T MRI Scanner (Siemens Espree) and 32 channel coil (Siemens Tim). Following a 10 minute rest period the same protocol was performed utilizing a standard dose of regadenoson (0.4mg IV over 10 seconds). A survey of side effects, medication tolerance and drug preference was given at the end of exam. All measurements were performed utilizing QMass (Vital Imaging, 2014).

## Results

At 5 minutes following infusion, adenosine had a more significant effect on biventricular volumes with up to a 20% increase seen when compared to regadenoson and continued to remain elevated at 15 minutes. Right and left ventricular ejection fractions increased by 5% at 15 minutes after adenosine infusion while the RVEF increased by 4% and LVEF reduced by 3% at 15 minutes following regadenoson dosing. Cardiac output immediately increased by 28% following adenosine while relatively unchanged by regadenoson. Both pulmonary arterial and aortic peak velocities were reduced on average of 0.5 m/sec at 5 minutes with each agent and returned to baseline values at 15 minutes. Heart rate and blood pressure returned to baseline with both agents at 15 minutes. Overall, study subjects preferred adenosine over regadenoson citing preference of adenosine's slow onset and rapid taper of side effects rather than the sudden onset and slow taper of side effects with regadenoson.

## Conclusions

Contrary to popular belief, both adenosine and regadenoson have significant and protracted effects on cardiac physiology despite return of vital signs to baseline. When compared to regadenoson, adenosine's effects on right and left ventricular volumes are much more significant yet both have minimal effect on outflow peak velocities. In the end, consideration should be given to timing of administration of each vasodilating agent and patient comfort when planning stress-CMR protocols.

## Funding

United States Air Force.

